# An unconquered challenge in MDS: review of pathophysiology, clinical manifestations, and management options of MDS with thrombocytopenia

**DOI:** 10.1007/s00277-025-06374-2

**Published:** 2025-09-12

**Authors:** Xiaoyi Chen, Mihir Shukla, Jun H. Choi

**Affiliations:** 1 Department of Medicine, Division of Hematology and Medical Oncology, New York University Grossman School of Medicine, New York, NY USA; 2 Division of Hematology and Medical Oncology, New York University Perlmutter Comprehensive Cancer Center, New York, NY USA; 3 Division of Blood Disorders, Cancer Institute of New Jersey, Rutgers University, East Brunswick, NJ USA; 4 Perlmutter Comprehensive Cancer Center, New York University School of Medicine, New York University Langone Health, 240 E 38th Street, 19th Floor, New York, NY 10016 USA

**Keywords:** Myelodysplastic syndrome, Thrombocytopenia, Romiplostim, Eltrombopag, Thrombopoietin

## Abstract

Myelodysplastic syndromes (MDS) is a heterogeneous group of myeloid clonal disorder resulting in bone marrow failure with a tendency to acute myeloid leukemia transformation. MDS is characterized by a variable degree of clonal cytopenia. Compared to anemia, thrombocytopenia is less common but presents more significant challenges due to high risk of acute complications and dearth effective treatment options. Platelet transfusions are effective in increasing platelet counts but provide limited and transient benefits, along with associated risks of transfusions. Anti-fibrinolytic drugs have been attempted including in clinical trial settings but its efficacy remains unproven. Successful development of thrombopoietin agonists appeared promising especially in other conditions associated with thrombocytopenia but its utility in MDS has been controversial. Two of the novel thrombopoietin receptor agonists (TPO-RA), romiplostim and eltrombopag have established clinical activity in immune thrombocytopenic purpura (ITP) and have been explored for the treatment of thrombocytopenia in MDS. Due to early research data showing TPO-RA leading to a small increase in blast counts and possibly promoting leukemic transformation, subsequent clinical trials sought to establish its safety and efficacy in MDS. Despite considerable amount of evidence demonstrating favorable safety profiles in lower risk MDS, many hematologists are often hesitant to use TPO-RA to treat thrombocytopenia in MDS due to theoretical concern of stimulating blasts. In higher risk MDS the safety is not proven and certainly requires more investigation. In this review, we aim to highlight pathophysiology of thrombocytopenia in MDS and provide comprehensive management strategies supported by past and current clinical research data.

## Introduction

Myelodysplastic neoplasms, or myelodysplastic syndromes (MDS), are a group of clonal hematopoietic neoplasms characterized by chronic cytopenias and morphologic dysplasia [1, 2]. Patients with MDS most commonly present with anemia, then with neutropenia or thrombocytopenia. The presence and severity of thrombocytopenia is a well-known prognostic indicator [3], with severe cases that can potentially lead to devastating complications and management challenges including life-threatening hemorrhage or inability to carry out necessary procedures or surgeries. Unlike anemia, for which more treatment options have been developed over the last decade, safe and effective therapy for thrombocytopenia in MDS treatment is still lacking. Platelet transfusion, a commonly used treatment strategy, is often associated with a variety of risks, such as alloimmunization, transfusion reaction, and risk of infection [4]. More recently, a notable development for treating thrombocytopenia is the introduction of various thrombopoietin (TPO)mimetics. Antibody based TPO receptor agonists such as romiplostim or small molecular based agent such as eltrombopag have been proven to be highly effective in increasing platelet counts in immune-thrombocytopenic purpura (ITP) and aplastic anemia [5, 6], but their role in MDS is less clear. The safety and the efficacy of TPO mimetics in MDS are being actively investigated in a number of clinical trials but mainly limited to lower risk low- or intermediate-1- risk groups by IPSS [7–11]. The main concerns are thrombogenicity and risk of blast stimulation, especially in intermediate- or high- risk MDS [12]. Other known treatments, such as hypomethylating agents, have not been proven to improve thrombocytopenia effectively [13]. This review aims to provide updated knowledge regarding the pathophysiology, clinical manifestations, and treatment of thrombocytopenia in MDS patients, and to summarize the present status and future directions of drug developments in this challenging population.

## Epidemiology

According to SEER database, the yearly incidence rate of MDS in 2022 is approximately 4 per 100 000 people [14]. It is more common in men (with yearly incidence rates of approximately 5.5 per 100 000 vs. 3 per 100 000 for women) and is more common among people who are non-Hispanic White (4.5 per 100 000) compared with people who are non-Hispanic Asian/Pacific Islander (2.6 per 100 000), non-Hispanic Black (3.1 per 100 000), or Hispanic (3 per 100 000) [14]. The estimated thrombocytopenia prevalence in MDS is about 40–65%, with a platelet count threshold of < 100 × 10^9^/L [15, 16]. Severe cases of thrombocytopenia, with cutoff platelet count < 20 × 10^9^/L, happens in about 7% of MDS patients in a study by Neunkirchen et al. [17], and 17% in a retrospective study from MD Anderson Cancer Center (MDACC) [16]. Although the incidence of severe thrombocytopenia in MDS varies among different studies, the trend that severe thrombocytopenia more often happens in higher risk MDS patients, correlates with severe bleeding complications and predicts worse prognosis is commonly observed. There are also about 5–10% of MDS patients with initial presentation of isolated thrombocytopenia [15], who are often misdiagnosed as ITP.

## Pathophysiology of thrombocytopenia in MDS

### Dysmegakaryopoiesis

MDS is characterized by dysplastic and ineffective hematopoiesis that leads to cytopenias with risks of transformation to acute myeloid leukemia (AML). With advances in research, studies have unraveled the pathophysiology of MDS in multiple levels including cellular, cytogenetic and molecular aspects. Studies of pathophysiology specific to thrombocytopenia in MDS also show a multilevel dysregulation of normal megakaryopoiesis. In MDS, megakaryocytes are often observed to be dysplastic with characteristic micromononuclear megakaryocytes, in which a dissociation of nuclear and cytoplasmic maturation is observed and a terminal differentiation of megakaryocytes is arrested [18]. 

### Thrombopoietin (TPO) signal pathway

A couple of signaling pathways have been identified that regulate normal megakaryopoiesis, among which the TPO signaling pathway is considered principal. TPO was purified and cloned by different research groups in 1994 [19–23], which made the last major hematopoietic growth factor identified historically. Binding of TPO to its receptor, c-Mpl, activates downstream JAK2 and STAT signaling pathways which stimulates megakaryocyte maturation and platelet production [24–26]. The circulating level of TPO is solely based on the rate of clearance via platelet and megakaryocyte, with the liver producing TPO at a constant rate [27]. In situations with liver disease or resection, TPO level decreases as well as the platelet counts. In conditions of reduced platelet production, such as post-chemotherapy periods, TPO level increases due to a decreased clearance rate, which in turn stimulates megakaryocytes to produce more platelets in a compensatory manner. In MDS, studies showed that the number of platelets and serum TPO levels were not simply reversely correlated as in physiological conditions, but varied depending on subtypes of MDS. For example, in MDS patients with refractory anemia (RA), TPO level reversely correlates with platelet counts, but not in RA patients with excess of blasts (RAEB), which could possibly due to the expression of c-Mpl in blasts [28, 29]. Due to the small sample size in these studies, it is difficult to draw conclusion about the correlation of platelet counts and TPO level in MDS. Nevertheless, the use of TPO mimetics to treat thrombocytopenia has been explored in lower risk MDS patients in clinical trials, with a response rate of 35–50% [7, 30, 31]. Unfortunately, safety issues also emerged, with a main concern of increased risk of leukemic transformation by using TPO mimetics, which will be further discussed in the *management* section.

### Additional signaling pathways

In addition to TPO, other cytokines have been found to affect megakaryocyte maturation, either in a TPO-dependent or independent manner. Cytokines that were found to affect megakaryopoiesis in a TPO-dependent manner include IL (interleukin) -1β [32], IL-3 [33], IL-6 [34], while IL-1α [35] and C-C motif chemokine ligand 5 (CCL5) [36] affect megakaryopoiesis in a TPO-independent manner, demonstrated by either in vivo or in vitro cell or animal studies. In addition, studies have also demonstrated the Notch pathway as an essential regulatory pathway during the normal process of megakaryopoiesis. The activity of Notch signaling changes according to different stages of megakaryocyte maturation, with an upregulation required for earlier progenitor development and a downregulation for terminal megakaryocyte comitment [37, 38]. Although studies have suggested a neatly regulated network involving growth factor, cytokines and signaling pathways in normal megakaryopoiesis, how these factors contribute to the pathophysiology of dysplastic megakaryopoiesis remains elusive.

### Genetic alternations

It is known that MDS is driven by a complex of recurrent genetic alternations that include both germline and somatic mutations [39]. Genes that are found to have roles in the disease pathogenesis involves multiple cellular processes such as epigenetic regulation, RNA splicing, transcriptional process, and signaling pathways [40]. Known gene mutations that are associated with thrombocytopenia in MDS include *ANKRD26*,* TP53*,* NRAS*,* RUNX1* and *ETV6* [41–44]. In 2016, the revision to world health organization (WHO) myeloid neoplasms (MNs) and acute leukemia classification described a class of MNs with germline predisposition and pre-existing platelet disorders where variants in *RUNX1*,* ANKRD26* and *ETV6* were included [45]. These gene variants were found associated with hereditary platelet disorders as well as significantly increased risk for myeloid neoplasm, including MDS [46]. Germline *RUNX1* variants is most commonly associated with life-long moderate thrombocytopenia and platelet dysfunction [47]. Mechanisms of how *RUNX1* variants lead to thrombocytopenia have been shown affecting multiple aspects of megakaryopoiesis, including megakaryocyte differentiation and maturation [48, 49]. *ETV6* is a transcription factor and *ETV6* knockout mice have thrombocytopenia with an increased megakaryocytic colonies, suggesting a role of *ETV6* in megakaryocyte terminal maturation [50]. ANKRD26 is an ankyrin-repeat domain protein and its persistent expression leads to thrombocytopenia via activating MAPK pathway [51]. 

### Clonal hematopoiesis

Since the observation and description of clonal hematopoiesis (CH) 1960s [52], rigorous studies in this field have helped us better understand both normal hematopoiesis and leukemogenesis. We now consider CH as a malignancy precursor, comparable to monoclonal gammopathy of unknown significant in multiple myeloma. The most common mutations observed in CH include *DNMT3A*, *TET2* and *ASXL1*, which overlaps with genetic mutations observed in MDS. The yearly rate of transformation to hematological neoplasia is 0.5–1% among people with CH, which is about 13 times higher than general population [53]. The mechanism of CH transformation to hematological malignancies including MDS are still largely unclear, but it is observed that chemotherapy and radiation are selection pressures that can drive selection and transformation of pre-existing CH, which largely expanded our current knowledge of secondary MDS [54]. Several features of CH associated with evolution to myeloid neoplasm have been identified. In particular, platelet of less than 100 × 10^9^/L was a significant risk factor of progression with a hazard ratio of 2.49, while other cytopenias did not show a major impact [55]. Researches of the evolutionary process in clonal hematopoiesis demonstrated genotype specific clonal dynamics, where, for example, *DNMT3A* clone grows faster at younger age, splicing factor mutated clones expand only in older age and a *TET2* mutated clone emerge across all ages [56]. Studies in clonal evolution will ultimately shed light on the mechanism of malignant transformation and help us understand the pathophysiology with varied phenotypes of MDS.

### Bone marrow microenvironment

Adult hematopoietic stem cells (HSCs) mainly reside in bone marrow niches, which is a local microenvironment that support and regulate stem cells. There are both cellular and non-cellular components in the niche that collectively participate in normal hematopoiesis. The cellular component is composed of cells such as endothelial cells, stromal cells, immune cells and osteocytes. The non-cellular component includes cytokines, extracellular matrix, and other soluble factors [57]. Studies have suggested that changes or abnormalities happening in the microenvironment may contribute to pathophysiology of MDS. For example, in mice, conditional knockout of *Dicer1*, an RNase III endonuclease essential for microRNA biogenesis, in osteoblastic lineage led to myelodysplasia, while *Dicer1* remains intact in hematopoietic cells [58]. A proper bone marrow environment is also required for thrombopoiesis. In the life cycle of megakaryocytes, they established interactions with multiple cell types from the bone marrow microenvironment which are necessary for their proper differentiation, maturation and platelet production. Additionally, a proper cross talk between megakaryocytes and circulating soluble regulatory factors and local extracellular matrix is necessary for normal platelet production [59, 60]. With advances in single cell research, where singe cells can be spatially traced and together with single cell dissection and sequencing, the future of understating roles of each type of cells in the bone marrow microenvironment in MDS pathophysiology and thrombocytopenia is promising [61, 62]. 

## Clinical diagnosis and classification of MDS

The very initial standard diagnosis criteria and classification system of MDS was described by French-British-American working group and was solely morphology based, including dysplasia, percentage of blasts, ring sideroblasts, monocytic proliferation and Auer rods [63]. About two decades later, the WHO classification system first incorporated karyotype abnormality into MDS classification, where del(5q) was recognized as an independent subclass [64]. It then took another two decades to add more MDS subclasses based on genetics. In 2022, both WHO 5th edition and the international consensus classification (ICC) introduced 2 new MDS entities: *SF3B1* mutation and multi-hit *P53* [1, 2], to classify MDS subtypes based on genetic alternations that has prognostic relevance, with *SF3B1* and del(5q) MDS having a relatively indolent clinical course, and *p53* mutated MDS being very aggressive and rapidly progressing. While the WHO 5th edition and ICC are largely similar, there are differences between the two classification systems which was compared in detail in literatures published prior [65, 66]. MDS with isolated thrombocytopenia are associated more favorable prognosis [67, 68]. Liapis et al. reported that del(20q) was the most frequent observed genetic abnormalities in MDS with isolated thrombocytopenia [68]. It is foreseeable that with the fast growing knowledge of MDS pathophysiology from research, the classification system with be more comprehensive and mechanistically based.

### Thrombocytopenia in risk stratification for MDS

Thrombocytopenia is a noted independent risk factor for poor prognosis in MDS. In an early large retrospective study, thrombocytopenia predicts worse prognosis across all international prognostic scoring system (IPSS) risk groups. In addition, severe thrombocytopenia was found correlating with poor performance, other cytopenias, advanced MDS phases and adverse karyotypes [69]. In MDS patients with thrombocytopenia, there is association between low platelet counts and significantly shortened survival, mainly due to progression to AML [17]. Decreasing platelet count after diagnosis also adversely affect prognosis across all risk categories of MDS. Patients who had 25% drop in platelets within the first 6 months after diagnosis had lower median survival (21 months versus 49 months, *p* < 0.001) and higher 2 year cumulative incidence of AML evolution (22% versus 8.3%, *p* < 0.001) [67]. Isolated thrombocytopenia, in contrast, is associated with better median overall survival compared to multi-cytopenia (109 months versus 55 months, *p* = 0.013), regardless of the severity of initial platelet decrease [68]. It is essential to carefully asses bone marrow aspirates and molecular profiles to prevent misdiagnosing them with ITP which can occur in up to 15% of cases in patients with MDS with isolated thrombocytopenia. Recognizing the severity of thrombocytopenia predicts prognosis, the revised-IPSS (IPSS-R) incorporated the degree of thrombocytopenia in risk stratifying patients. In the most recent molecular-IPSS (IPSS-M), the degree of thrombocytopenia remains an independent risk factor [70]. In other risk stratification systems, such as WHO prognostic scoring system (WPSS), FCC/MLL, and EuroMDS, platelet count is included as an independent prognostic factor, solidifying the importance of thrombocytopenia in predicting MDS prognosis [71–73]. 

### Clinical manifestation of thrombocytopenia in MDS

The clinical symptoms of thrombocytopenia vary from asymptomatic thrombocytopenia to severe life-threatening bleeding. In a large retrospective study, thrombocytopenia was found to be the third leading cause of death and taking up 9.8% of disease related mortality, following leukemia transformation (46.6%) and infection (27%) [74]. A study from MDACC also reported that hemorrhages, as the only cause, accounted for 10% of the death in MDS [16]. It is noted that in MDS patients at diagnosis, about 30% had bleeding events even with a relatively high platelet counts, suggesting platelet malfunction [67]. The most common platelet dysfunction in MDS is decreased or lack of platelet aggregation. In a small cohort study with 21 MDS patients, majority (81%) of patients’ platelets had impaired platelet aggregation in response to stimulants [75]. In another comprehensive proteomic analysis, proteins, which are critical to integrin α_IIb_β_3_ signaling pathway and thus platelet aggregation, were found at a lower concentration in MDS patients, providing molecular explanation for platelet aggregation dysfunction [76]. The dysfunction of platelet adds challenge to clinical practice when deciding individual appropriated platelet transfusion threshold and also making it more difficult to predict risk of bleeding if solely relying on platelet counts.

## Management of thrombocytopenia in MDS

### Platelet transfusion

Platelet transfusion is a reliable and effective therapy to rapidly restore platelet counts. It could be lifesaving treatment especially in patients with severe active bleeding. However, platelet transfusion is related to a number of risks and limited by persistent blood product shortage. Unlike other blood products, platelets need to be stored at room temperature to preserve its function, which leads to increased risk of bacterial contamination that can cause life-threatening sepsis in recipients. It is estimated that between 1:1000 and 1:2500 platelet units are bacterially contaminated with sources largely coming from skin flora that contaminate platelet during collection [77]. Although strategies, such as donor selection, culture testing, rapid molecular tests and UV lights to inactive pathogens have been used to improve platelet safety. The risk of platelet contamination and transfusion related infection cannot be fully eliminated [77]. 

Platelet alloimmunization is another risk for MDS patients who usually require recurrent platelet transfusion and consequently form alloantibodies in response to foreign antigens. It is estimated that platelet alloimmunization occurred in about 20 to 85% of patients who have received multiple transfusions [78]. Platelets express a variety of antigens which can be divided into two main categories: (1) platelet specific antigen such as glycoproteins and human platelet antigens and (2) common antigens that share with other cells such as ABO and human leukocyte antigen (HLA) class I. Alloimmunization to HLA class I represents the most common immunological cause of platelet transfusion refractoriness [79]. Once alloantibodies are formed, the management is very challenging. An HLA-matched or -selected and crossmatched platelets is the main evidence-based treatment. However, this often requires longer waiting period for the matched product with increased risk of bleeding, as well as a higher financial burden. Additionally, a matched platelet is effective in improving platelet counts at 1-hour post post-transfusion, but it is unclear if this is will lead to decreased bleeding rates or mortality [79]. 

Additional issues related to platelet transfusion, such as transfusion related febrile response, short shelf life of platelet products (less than 7 days), short circulating half-life of platelets (2 to 3 days) and transfusion requiring hospital setting, all point to the fact that platelet transfusion is not the ideal therapy for MDS with thrombocytopenia and alternative treatments are urgently needed [78]. Furthermore, a shortage in platelet supply, as well as other blood products, has been historically worsening. In addition, prophylactic transfusion of platelets do not often result in significant decrease in bleeding events in MDS patients [80]. According to American Red Cross, the number of people who are donating is at an all-time low for the past 20 years, making platelet transfusion even more challenging [81]. 

### Anti-fibrolytic agent

An inhibitor of fibrinolysis such as aminocaproic acid or tranexemic acid (TXA) has been used to both prevent and treat bleeding in thrombocytopenic patient. TXA is a lysine analogue that is a competitive inhibitor of plasminogen activation and, at higher concentrations, a non-competitive inhibitor of plasmin [82]. There is no prospective randomized trial assessing the efficacy of TXA in MDS patients with thrombocytopenia but several small retrospective studies and physician surveys provide its potential utility. A single center observational registry study showed that among 99 patients with severe persistent thrombocytopenia, 67 patients were treated with TXA, either alone (28%) or with prophylactic platelet transfusions (39%). Grade 3–4 bleeding events occurred in 6% of patients. Although major bleeding was uncommon, there were no significant differences in the rate of bleeding between the TXA treated or untreated patients [83]. In another retrospective study investigating predictors of bleeding in thrombocytopenic MDS patients, TXA use nor prophylactic platelet transfusion did not attenuate the rate of major bleeding events, which occurred in 11% of patients [80]. A large randomized controlled trial has been conducted evaluating the safety and efficacy of TXA in patients with hematologic malignancies with severe thrombocytopenia. Prophylactic TXA had no effect on the incidence of grade > = 2 bleeding or death when used in addition to prophylactic platelet transfusion. However, given that these patients underwent intensive chemotherapy or hematopoietic stem cell transplants, the conclusion cannot be easily extrapolated for MDS patients with pre-existing thrombocytopenia. In a clinician practice survey to analyze the real world management of MDS-related thrombocytopenia, nearly all respondents (94%) responded that they would consider prescribing TXA for MDS patients but most (91%) did not have institutional guidelines, underlying the need for future prospective randomized control study [84]. 

### Thrombopoietic mimetics

TPO signaling pathway is the main regulatory pathway for platelet production. Since the discovery of TPO in the 1990s, researchers have been exploring the possibility of targeting this signaling pathway to stimulate platelet production in thrombocytopenic patients. The first generation TPO receptor agonists (TPO-RA), pegylated recombinant human megakaryocyte growth and development factor, were able to increase platelet counts in both healthy and thrombocytopenic patients. However, clinical studies were later terminated due to thrombocytopenia in healthy volunteers secondary to formation of antibodies towards TPO-RA that cross-reacts with endogenous TPO [85]. Second generation TPO-RA were developed with a focus of minimizing structural similarities and are represented by TPO peptide mimetics and non-peptide small molecules. Romiplostim represents small peptide mimetics which is given subcutaneously to activate TPO receptor by binding to the distal hematopoietic receptor domain similar to endogenous TPO. Eltrombopag is a small molecule mimetic that activates TPO receptor via binding to its transmembrane domain [86]. Both romiplostim and eltrombopag were approved for ITP treatment after proven to be effective in raising platelet counts in clinical trials [87, 88]. Two additional small molecules that target the same site of TPO receptors as eltrombopag were also FDA approved in clinical use: avatrombopag for both ITP and thrombocytopenia of chronic liver disease and lusutrombopag for thrombocytopenia of chronic liver disease [89, 90]. A third class of TPO-RA, represented by TPO receptor antibodies, is also being investigated and has shown promising effects in chemotherapy related thrombocytopenia in mouse models [91]. 

### TPO mimetics in lower risk MDS

Both romiplostim and eltrombopag have been studied for their efficacy and safety in MDS patients with thrombocytopenia in randomized clinical trials. They were used as either single treatment or in combination with chemotherapy. Kantarjian et al. reported a multi-center, phase I/II dose-escalation trial, where romiplostim was used as a single agent in IPSS low- or intermediate-1-risk MDS patients at different doses weekly as subcutaneous injection. A total of 44 patients completed the treatment phase (4 weeks) and 41 patients continued into the extension phase (up to one year of romiplostim treatment). Results showed that median platelet counts increased across different dose groups and 19 (46%) patients achieved a durable response. There were fewer bleeding events and decreased platelet transfusion needs among patients who achieved a durable response than those who did not (4.3 v 39.3 per 100 patient-weeks). During the study, two patients progressed to acute myeloid leukemia [92]. Subsequently, a double blind, multi-center phase II clinical trial recruited 250 low- or intermediate-1-risk MDS patients and randomized 2:1 to either single romiplostim or placebo treatment. Studied was designed to give romiplostim injection weekly for 58 weeks with a primary end point of the number of clinically significant bleeding events (CSBEs) per patient. CSBEs was significantly reduced in patients with a baseline platelet counts more than 20 × 10^9^/L (*P* < 0.0001), but not in patients with a baseline platelet counts less than 20 × 10^9^/L. Platelet transfusion rate was higher in placebo group with a *P* value less than 0.0001. However, the study was terminated based on the interim analysis due to the concern for excess blasts and AML transformation rates in romiplostim treated arm with a HR of 2.5. At 58 weeks, the rate of progression to AML was 6% in the romiplostim arm and 4.9% in the placebo arm with a HR of 1.2 and 95% confidence interval (CI) of 0.38 to 3.84 [30]. Although romiplostim treatment in the trial was discontinued, patients remained on study for follow-up. A 5-year long-term follow up of 210/250 (84%) patients showed that 20 (12%) in romiplostim group vs. 9 (11%) in the placebo group progressed to AML with a HR of 1.06 (95% CI 0.58–2.33) and 93 (56%) vs. 54 (54%) patients died, respectively, with a HR of 1.03 (95% CI 0.72–1.47) [31]. The EUROPE phase 2 clinical trial was designed to investigate the predictive value of biomarkers when using romiplostim in lower risk MDS patients with thrombocytopenia. In this study, thirty-two out of 77 patients (42%) achieved hematological improvement of platelets with a median increasement of 47 /nL and a median duration of 340 days. There was no increased rate of leukemic progression observed during treatment [93]. In addition to single agent treatment, romiplostim was also studied in combination with chemotherapies in randomized clinical trials (Table 1). The chemotherapies that were used in combination were lenalidomide [94], decitabine [95] or azacitidine [96]. These were all small trials with about 20 to 40 patients recruited in each trial. It is difficult to draw any solid conclusions yet with the limitation of small sample size and large randomized clinical trials are needed to validate clinical benefits and safety of romiplostim for the future.

Eltrombopag was also tested for its safety and efficacy in treating severe thrombocytopenia in lower risk MDS. Vincent et al. reported a phase II dose modification study, where eltrombopag was initiated as a single agent at 50 mg daily with dose escalation to a maximum of 150 mg daily over 16 weeks in lower risk MDS patients with cytopenia. Eleven of 25 patients (44%) met primary endpoint who had hematologic response at 16–20 weeks. Six out of eleven (55%) responders had platelet improvement. In safety analysis, there was no serious adverse events attributed to eltrombopag at data collection [97]. EQOL-MDS is a phase II, single-blind, placebo-controlled trial, in which a total of 169 low- or intermediate-1-risk MDS patients with thrombocytopenia were randomized 2:1 to eltrombopag or placebo treatment till disease progression. The primary end point was duration of platelet response (PLT-R), long-term safety and tolerability. The recently published 60 months long-term follow up results showed that PLT-R is significantly higher in eltrombopag arm (43.2%) compared to placebo (11.1%) with a OR of 5.9 (95% CI 2.3–14.9, *P* < 0.001). Clinically significant bleeding was significantly less in the eltrombopag arm than in the placebo group (incidence rate ratio, 0.54; 95% CI, 0.38 to 0.75; *P* = 0.0002). AML evolution and/or disease progression were similar (17%) for both eltrombopag and placebo arms and a similar survival time was also observed [9]. In a retrospective study, Comont et al. [98] reported the real world use of eltrombopag in low-risk MDS (50 patients) and chronic myelomonocytic leukemia (CMML, 11 patients) patients with platelet count < 50 × 10^9^/L. Forty-seven (77%) out of 61 patients reached a platelet response with a median duration of 8 (0–69) months. Disease progression was observed in ten patients, which was comparable to historical non-TPO treated cohort and suggested natural evolution of disease and safe to use eltrombopag in this group of patients. With different directions of trial results (Table 1), it is still unclear about the appropriate clinical scenario for eltrombopag usage. However, it appears safe and effective in lower risk MDS with thrombocytopenia patients.

### TPO mimetics in higher risk MDS

As eltrombopag targets a different site on TPO-R than romiplostim, it also behaves differently with an anti-leukemic effect in preclinical researches. Various mechanisms have been proposed to explain the anti-leukemic effect of eltrombopag. Roth et al. found that eltrombopag was able to chelate iron and thus decrease the intracellular iron level, which subsequently led to cell cycle arrest and leukemia cell differentiation. Increasing the intracellular level abrogates eltrombopag-mediated antiproliferation and differentiation inducing effects [99]. Eltrombopag was also found to be able to rapidly decrease reactive oxygen species and cause cell cycle arrest and AML cell death [100]. The research findings of anti-leukemic effects provide a rational to test eltrombopag for MDS or AML treatment. In a pilot study, Svensson et al. reported that a maximum of 200 mg daily eltrombopag was well tolerated in combination with azacitidine in high-risk MDS patients. Nine out of twelve patients achieved stable or improved platelet counts. No increased blast counts or disease progression was observed [101]. A phase III, double-blind, multicenter SUPPORT clinical trial studied platelet supportive effect of eltrombopag in azacitidine treated MDS patients. A total of 356 intermediate- and high-risk MDS patients with platelet counts < 75 × 10^9^/L were randomized 1:1 to eltrombopag or placebo in combination with azacitidine treatment. The primary end point was the rate of platelet transfusion independence. This study was terminated early due to futility and safety concern. At the second interim analysis, there was no difference in hematological improvement. There was also a trend toward an increase in the rate of progression to AML in the eltrombopag plus azacitidine arm, although it was not statistically significant [12]. Efficacy and safety of using eltrombopag as single agent treatment in MDS/AML were also explored in clinical trials. Platzbecker et al. reported a phase I/II randomized, placebo-controlled, double-blind trial where eltrombopag were used as a single agent in high-risk MDS or AML patients who had platelet counts less than 30 × 10^9^/L or platelet transfusion dependent. In the trial, 98 patients were randomized 2:1 to eltrombopag or placebo. Primary end point was maximum tolerated eltrombopag dose and safety. Results showed that a maximum of 300 mg/day eltrombopag was tolerated and there was no difference in increasing median bone marrow or peripheral blast counts between two groups [102]. ASPIRE trial is another phase II randomized, double-blind clinical trial, in which the efficacy of eltrombopag in reducing clinically relevant thrombocytopenic events (CRTE) was evaluated. A total of 145 high-risk MDS or AML patients with platelet counts < 25 × 10^9^/L or platelet transfusion dependency were randomized to eltrombopag or placebo arms. Results showed that average weekly CRTE was significantly lower with eltrombopag (54%) than with placebo (69%) and an odds ratio of 0.2 (95% CI 0.05 to 0.87, *P* = 0.032). The study did not show increased blast count, MDS progression or clinical worsening of leukemia in eltrombopag treated group [103].

### Hypomethylating agents

There are three hypomethylating agents (HMA), including azacitidine, decitabine, decitabine-cedazuridine, that have been approved by Food and Drug Administration (FDA) for adult MDS treatment. Both azacitidine and decitabine are analogs to nucleoside cytidine. Decitabine-cedazuridine is given orally with cedazuridine serving as a deaminase inhibitor that prevents decitabine from being metabolized in the GI tract [104]. After cellular uptake, both azacitidine and decitabine are metabolized into their active forms: 5-azacitdine-triphosphate for azacitidine and 5-aza-2’-deoxycytidine-triphosphate for decitabine. These active metabolites can directly incorporate into DNA (and mainly RNA for azacitidine) to cause DNA and/or RNA damage in MDS cells. They can also bind and inhibit DNA methyltransferase which leads to DNA hypomethylation that allows gene expression, including critical tumor suppressor genes. Additional mechanisms, such as impairing DNA repair, inducing apoptosis through disrupting rRNA processing, inducing immune responses in cancer cell and suppressing NF-Kappa B signaling pathways, were reviewed in detail in previously published literature [105]. 

MDS patients with thrombocytopenia are more likely to be in intermediate- or high-risk groups as described above and thus usually are treated with first-line hypomethylating agents. In randomized clinical trials, both azacitidine and decitabine demonstrated a less than 20% complete remission rate [106, 107]. In the azacitidine trial, a grade 3 or 4 thrombocytopenia occurred in 84% patients in azacitidine group, and 74% patients with grade 0 to 2 thrombocytopenia progressed to grade 3 or 4 during treatment [106]. Retrospective studies reported that early platelet response to decitabine treatment, was an independent predictor of better overall survival [108]. However, this was not proven to be true in mouse models where an early increment of platelet count was not observed after HMA treatment [109]. A recent randomized clinical trial used CC-486, oral azacitidine approved in AML post-remission therapy, in lower risk MDS showed a higher platelet response (24.3% vs. 6.5%) in comparison to placebo, while grade 3 or 4 thrombocytopenia occurred more commonly in CC-486 arm (29% vs. 15.6%) [110]. None of the HMA are approved specifically for the treatment of MDS with thrombocytopenia. A high incidence of grade 3 or 4 thrombocytopenia from HMA treatment can further complicate clinical management of MDS with pre-existing thrombocytopenia. In recent trials on high risk MDS patients, adding venetoclax to HMA therapy yielded superior response compared to historical data on HMA alone, with the CR rate of 30–50%. Packed red blood cell or platelet transfusion independence was achieved in approximately 40% of patients both in treatment naïve and treatment refractory MDS patients [111]. The combination therapy of HMA plus venetoclax will likely become the standard of therapy for high risk MDS patients pending results from the ongoing phase 3 study (NCT04401748).

## Discussion

Thrombocytopenia is a common yet challenging clinical scenario in MDS management as there is still no effective treatment. MDS patients with thrombocytopenia more commonly present with higher risk disease and a poor prognosis. TPO-RA was considered a promising treatment for MDS related thrombocytopenia. However, large sample sized clinical trial for both romiplostim and eltrombopag was terminated due to concern for the risk of leukemia transformation. Data seems more encouraging in lower risk MDS patients with single agent eltrombopag treatment which showed decreased bleeding events and platelet response, while an increased risk of AML transformation was not observed. Accumulating studies decribed in the earlier section support the safety and efficacy of using TPO mimetics in selected group of lower risk MDS with refractory thrombocytopenia (Table 1). Although there is no direct comparison of safety and efficacy between eltrombopag and romiplostim, some studies report more significant reduction of grade > = 3 bleeding events with eltrombopag compared to romiplostim [112]. 

Its safety in higher-risk MDS is still under investigation. A meta-analysis reviwing 8 trials involving TPO mimetics use in MDS reported a significant decrease in the overall response rate (ORR) in TPO-RA group compared with placebo, with a pooled risk ratio (RR) rate of 0.65 (*p* = 0.01). Particularly, the subgroup analysis revealed a significant difference for ORR in intermediate- or high-risk MDS (RR 0.65, *p* = 0.006) [112]. Based on these results, routine use of TPO-RAs in higher risk MDS patients should be avoided until further exploration.

**Fig. 1:**
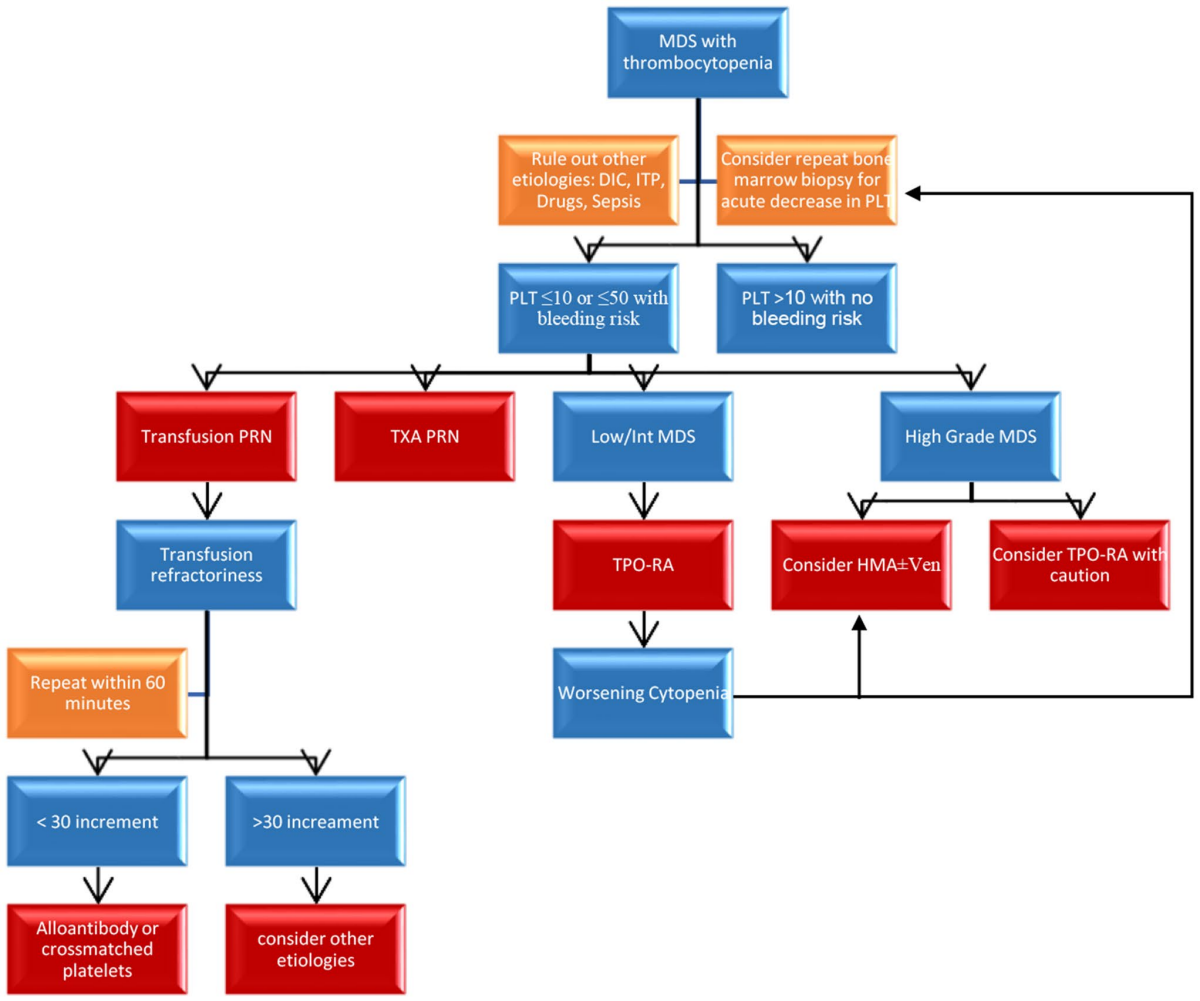
Management of thrombocytopenia in MDS

Prior to using TPO-RA, other more accessible management strategies should be exhausted. The first step is to ensure proper diagnosis is made and rule out other etiologies that can lead to thrombocytopenia. More common causes of thrombocytopenia include pseudothrombocytopenia, drug induced antibodies, infection, splenic sequestration, and disseminated intravascular coagulopathy (DIC). Not all thrombocytopenia in MDS patients require treatment but only in those with severe thrombocytopenia less than 10–20 /10^9^L or high risk of bleeding with less than 50/10^9^L. In patients who are not actively receiving cytotoxic treatment, occasional as-needed transfusions of apheresed platelet units may suffice. Use of anti-fibrolytic agent such as TXA can be considered but its efficacy has not been adequately tested. Risk of platelet transfusion refractoriness rises as the number of transfusions increases, partially due to potential alloimmunization. In order to establish platelet transfusion refractoriness, platelet levels should be rechecked within 10–60 min post-transfusions. If platelet count increment is less than 10/10^9^L, HLA- and HPA-typing can be considered to screen for allo-antibodies and request for crossmatched or antibody matched platelet products [113]. Any acute changes in platelet counts should lead to re-evaluation of bone marrow to rule out progression of existing MDS. For low to intermediate grade MDS, romiplostim or eltrombopaq can be utilized given sufficient evidence of tolerability and low risk of MDS progression. For high intermediate or high risk MDS, TPO-RA should be used more cautiously especially in cases with higher percentage of blasts, as the risk of blast stimulation is relatively higher. Although bleeding risk can be lowered and eltrombopag may have presumptive anti-leukemia effect, the lower ORR in TPO-RA treated patients compared to placebo in higher grade MDS patients is concerning. Chemotherapy such as HMA or lenalidomide should be restricted to higher grade MDS patients or to lower grade MDS patients with refractory thrombocytopenia despite using above mentioned treatment options, including TPO-RA (Fig. 1).

To date, there remains no curative therapy for MDS patients with thrombocytopenia except bone marrow transplant, which is a high-risk procedure with strict selection criteria and a large proportion of patients not qualifying for the treatment. In MDS with anemia, we have seen a prospering of drug development with better understanding of disease pathophysiology. Similarly, a better understanding of thrombocytopenia pathophysiology, such as regulations of megakaryopoiesis, mechanisms of platelet dysfunction, underlying difference across TPO-R agonists and mechanisms of TPO-RA-drug interactions, will provide opportunities optimal therapy combination and new drug development.

**Table 1:** Clinical trials with thrombopoietic agents in MDS with thrombocytopenia

Studies	Agents	Study details	Outcomes
Kantarjian et al. [97]	Romiplostim	Phase I/II 44 low- and intermediate-1-risk MDS patient with mean baseline platelets less than 50 K	Durable platelet response in 19 patients. Two patients transformed to AML during the study.
Giagounidis et al. [30]	Romiplostim	Phase II 250 low- or intermediate − 1-risk MDS patients with median platelet counts slightly lower than 20 K randomized 2:1 to romiplostim or placebo	Study was terminated early due to concern for excess blast and AML transformation
Kubasch et al. [98]	Romiplostim	Phase II 77 Lower risk MDS patients with a median platelet count of 25/nL were included. Romiplostim was started at 750 mcg weekly.	A hematologic improvement of platelet was observed in 32 (42%) out of 77 patients. No new safety concern and no increased leukemic progression were observed.
Kantarjian et al[101]	Romiplostim + Azacitidine	Phase II 40 low- or intermediate-risk patients were randomized 1:1:1 to romiplostim 500 mcg, 750 mcg or placebo during 4 cycles of azacitidine	Incidence of clinically significant thrombocytopenic events and platelet transfusion were not statistically different among groups due to small sample size.
Greenberg et al. [100]	Romiplostim + Decitabine	Phase II 29 low- or intermediate-risk patients randomized to 1:1 to romiplostim or placebo during 4 cycles of decitabine.	Study not powered to detect clinically significant thrombocytopenic events although platelet counts trended higher in romiplostim arm. No new safety concern or increase AML transformation with romiplostim treatment.
Wang et al. [99]	Romiplostim + Lenalidomide	Phase II 39 patients were randomized 1:1:1 to romiplostim 500 mcg, 750 mcg or placebo during 4 cycles of lenalidomide with an optional open-label extension period.	Study not powered to detect statistical different, although there was a trend toward higher median platelet counts in romiplostim groups. Two patients had increased bone marrow blast counts during treatment.
Platzbecker et al[109]	Eltrombopag	Phase I/II 98 high risk or AML patients were randomized 2:1 to receive eltrombopag or placebo.	Maximal tolerated dose of 300 mg qd. No increased risk in leukemia transformation in eltrombopag treated group
Mittelman et al. [110]	Eltrombopag	Phase II 145 high-risk MDS or AML patients randomized to eltrombopag or placebo.	Eltrombopag treatment significantly reduced clinically relevant thrombocytopenic events. No increased MDS progression of AML worsening in eltrombopag treated arm.
Vicent et al. [102]	Eltrombopag	Phase II 30 low or intermediate risk MDS patients with cytopenia was enrolled with a dose escalation from 50 mg daily to 150 mg daily. First five patients not included in efficacy analysis.	Eltrombopag at dose 150 mg daily was well tolerated. Eleven of 25 (44%) patients achieved hematologic response. No eltrombopag related death, thrombotic events, or progression to AML were observed at data cut off point.
Oliva et al. [9]	Eltrombopag	Phase II 169 low- or intermediate-1-risk MDS patients were randomized 2:1 to eltrombopag vs. placebo	Platelet response significantly higher in eltrombopag treated group. Clinically significant bleeding occurred significantly less frequently in eltrombopag treated group. No difference of AML transformation between two groups.
Svensson et al. [107]	Eltrombopag + Azacitidine	Pilot phase I 12 intermediate-2 or high-risk patient with a platelet < 75 K were included Eltrombopag was	Maximal tolerated dose of eltrombopag was 200 mg qd in combination with azacitidine. CR in 4/12 patients. platelet counts improved or remained stable in 9/12 patients.
Dickinson et al. [12]	Eltrombopag + Azacitidine	Phase III 356 intermediate or high-risk patients randomized 1:1 to receive eltrombopag vs. placebo in combination with azacitidine	Early termination due to futility and trend of AML transformation in eltrombopag + azacitidine group.

## Data Availability

No datasets were generated or analysed during the current study.

## References

[1] Khoury JD, Solary E, Abla O et al (2022) The 5th edition of the World Health Organization Classification of Haematolymphoid Tumours: Myeloid and Histiocytic/Dendritic Neoplasms. Leukemia. 36(7):1703–1719. 10.1038/s41375-022-01613-1 10.1038/s41375-022-01613-1 PMC9252913 35732831

[2] Hasserjian RP, Orazi A, Orfao A, Rozman M, Wang SA (2023) The international consensus classification of myelodysplastic syndromes and related entities. Virchows Arch 482(1):39–51. 10.1007/s00428-022-03417-1 36287260 10.1007/s00428-022-03417-1

[3] Greenberg PL, Tuechler H, Schanz J et al (2012) Revised international prognostic scoring system for myelodysplastic syndromes. Blood 120(12):2454–2465. 10.1182/blood-2012-03-420489 22740453 10.1182/blood-2012-03-420489 PMC4425443

[4] Kiefel V (2008) Reactions induced by platelet transfusions. Transfus Med Hemother 35(5):354–358. 10.1159/000151350 21512624 10.1159/000151350 PMC3076327

[5] Newland A, Godeau B, Priego V et al (2016) Remission and platelet responses with Romiplostim in primary immune thrombocytopenia: final results from a phase 2 study. Br J Haematol 172(2):262–273. 10.1111/bjh.13827 26537623 10.1111/bjh.13827

[6] Tichelli A, De Latour RP, Dufour C, Rovo A (2022) Adding Eltrombopag to immunosuppression: the importance of predicting outcome. Haematologica 107(1):46–48. 10.3324/haematol.2021.278761 33910335 10.3324/haematol.2021.278761 PMC8719093

[7] Oliva EN, Alati C, Santini V et al (2017) Eltrombopag versus placebo for low-risk myelodysplastic syndromes with thrombocytopenia (EQoL-MDS): phase 1 results of a single-blind, randomised, controlled, phase 2 superiority trial. Lancet Haematol 4(3):e127–e136. 10.1016/S2352-3026(17)30012-1 28162984 10.1016/S2352-3026(17)30012-1

[8] Oliva EN, Alati C, Santini V et al (2019) Long term effects of Eltrombopag treatment versus placebo for Low-Risk myelodysplastic syndromes with thrombocytopenia (EQoL-MDS): interim results of a Single-Blind, randomised, controlled, phase 2 superiority trial. Blood 134(Supplement1):3000–3000. 10.1182/blood-2019-126744

[9] Oliva EN, Riva M, Niscola P et al (2023) Eltrombopag for Low-Risk myelodysplastic syndromes with thrombocytopenia: interim results of a phase II, randomized, Placebo-Controlled clinical trial (EQOL-MDS). J Clin Oncol 41(28):4486–4496. 10.1200/JCO.22.02699 37294914 10.1200/JCO.22.02699 PMC10552995

[10] Fenaux P, Muus P, Kantarjian H et al (2017) Romiplostim monotherapy in thrombocytopenic patients with myelodysplastic syndromes: long-term safety and efficacy. Br J Haematol 178(6):906–913 (In eng). 10.1111/bjh.14792 28616874 10.1111/bjh.14792 PMC5600084

[11] Kantarjian H, Fenaux P, Sekeres MA et al (2016) Romiplostim in thrombocytopenic patients with Low- or Int-1- risk MDS results in reduced bleeding without impacting leukemic progression: final Follow-up results from a randomized, Double-Blind, Placebo-Controlled study. Blood 128(22):2000–2000. 10.1182/blood.V128.22.2000.2000

[12] Dickinson M, Cherif H, Fenaux P et al (2018) Azacitidine with or without Eltrombopag for first-line treatment of intermediate- or high-risk MDS with thrombocytopenia. Blood 132(25):2629–2638. 10.1182/blood-2018-06-855221 30305280 10.1182/blood-2018-06-855221 PMC6337824

[13] Yang J, Choi N, Paulson K et al (2022) Evaluation of bleeding and thrombocytopenia in patients with myelodysplastic syndromes treated with hypomethylating agents: A systematic review. Blood 140(Supplement 1):12315–12316. 10.1182/blood-2022-170553

[14] SEER*Explorer: An interactive website for SEER cancer statistics [Internet]. Surveillance Research Program, National Cancer Institute (2023) Apr 19. [updated: 2023; cited 2024 Jan 8]. Available from: https://seer.cancer.gov/statistics-network/explorer/. Data source(s): SEER Incidence Data, November 2022 Submission (1975–2020), SEER 22 registries

[15] Steensma DP, Bennett JM (2006) The myelodysplastic syndromes: diagnosis and treatment. Mayo Clin Proc 81(1):104–130. 10.4065/81.1.104 16438486 10.4065/81.1.104

[16] Kantarjian H, Giles F, List A et al (2007) The incidence and impact of thrombocytopenia in myelodysplastic syndromes. Cancer 109(9):1705–1714. 10.1002/cncr.22602 17366593 10.1002/cncr.22602

[17] Neukirchen J, Blum S, Kuendgen A et al (2009) Platelet counts and haemorrhagic diathesis in patients with myelodysplastic syndromes. Eur J Haematol 83(5):477–482. 10.1111/j.1600-0609.2009.01299.x 19548919 10.1111/j.1600-0609.2009.01299.x

[18] Houwerzijl EJ, Blom NR, van der Want JJ, Vellenga E, de Wolf JT (2006) Megakaryocytic dysfunction in myelodysplastic syndromes and idiopathic thrombocytopenic purpura is in part due to different forms of cell death. Leukemia 20(11):1937–1942. 10.1038/sj.leu.2404385 16990774 10.1038/sj.leu.2404385

[19] de Sauvage FJ, Hass PE, Spencer SD et al (1994) Stimulation of megakaryocytopoiesis and thrombopoiesis by the c-Mpl ligand. Nature 369(6481):533–538. 10.1038/369533a0 8202154 10.1038/369533a0

[20] Lok S, Kaushansky K, Holly RD et al (1994) Cloning and expression of murine thrombopoietin cDNA and stimulation of platelet production in vivo. Nature 369(6481):565–568. 10.1038/369565a0 8202158 10.1038/369565a0

[21] Bartley TD, Bogenberger J, Hunt P et al (1994) Identification and cloning of a megakaryocyte growth and development factor that is a ligand for the cytokine receptor Mpl. Cell 77(7):1117–1124. 10.1016/0092-8674(94)90450-2 8020099 10.1016/0092-8674(94)90450-2

[22] Kuter DJ, Beeler DL, Rosenberg RD (1994) The purification of megapoietin: a physiological regulator of megakaryocyte growth and platelet production. Proc Natl Acad Sci U S A 91(23):11104–11108. 10.1073/pnas.91.23.11104 7972018 10.1073/pnas.91.23.11104 PMC45175

[23] Kato T, Ogami K, Shimada Y et al (1995) Purification and characterization of thrombopoietin. J Biochem 118(1):229–236. 10.1093/oxfordjournals.jbchem.a124883 8537317 10.1093/oxfordjournals.jbchem.a124883

[24] Sattler M, Durstin MA, Frank DA et al (1995) The thrombopoietin receptor c-MPL activates JAK2 and TYK2 tyrosine kinases. Exp Hematol 23(9):1040–1048. https://www.ncbi.nlm.nih.gov/pubmed/7543416 7543416

[25] Tortolani PJ, Johnston JA, Bacon CM et al (1995) Thrombopoietin induces tyrosine phosphorylation and activation of the Janus kinase, JAK2. Blood. 85(12):3444-51. (https://www.ncbi.nlm.nih.gov/pubmed/7780132) 7780132

[26] Bacon CM, Tortolani PJ, Shimosaka A, Rees RC, Longo DL, O’Shea JJ (1995) Thrombopoietin (TPO) induces tyrosine phosphorylation and activation of STAT5 and STAT3. FEBS Lett 370(1–2):63–68. 10.1016/0014-5793(95)00796-c 7544303 10.1016/0014-5793(95)00796-c

[27] Yang C, Li YC, Kuter DJ (1999) The physiological response of thrombopoietin (c-Mpl ligand) to thrombocytopenia in the rat. Br J Haematol 105(2):478–485. https://www.ncbi.nlm.nih.gov/pubmed/10233424 10233424

[28] Tamura H, Ogata K, Luo S et al (1998) Plasma thrombopoietin (TPO) levels and expression of TPO receptor on platelets in patients with myelodysplastic syndromes. Br J Haematol 103(3):778–784. 10.1046/j.1365-2141.1998.01054.x 9858230 10.1046/j.1365-2141.1998.01054.x

[29] Zwierzina H, Rollinger-Holzinger I, Nuessler V, Herold M, Meng YG (1998) Endogenous serum thrombopoietin concentrations in patients with myelodysplastic syndromes. Leukemia 12(1):59–64. 10.1038/sj.leu.2400901 9436921 10.1038/sj.leu.2400901

[30] Giagounidis A, Mufti GJ, Fenaux P et al (2014) Results of a randomized, double-blind study of Romiplostim versus placebo in patients with low/intermediate-1-risk myelodysplastic syndrome and thrombocytopenia. Cancer 120(12):1838–1846. 10.1002/cncr.28663 24706489 10.1002/cncr.28663 PMC4298760

[31] Kantarjian HM, Fenaux P, Sekeres MA et al (2018) Long-term follow-up for up to 5 years on the risk of leukaemic progression in thrombocytopenic patients with lower-risk myelodysplastic syndromes treated with Romiplostim or placebo in a randomised double-blind trial. Lancet Haematol 5(3):e117–e126. 10.1016/S2352-3026(18)30016-4 29396092 10.1016/S2352-3026(18)30016-4

[32] Kimura H, Ishibashi T, Shikama Y et al (1990) Interleukin-1 beta (IL-1 beta) induces thrombocytosis in mice: possible implication of IL-6. Blood 76(12):2493–2500. https://www.ncbi.nlm.nih.gov/pubmed/2265245 2265245

[33] Segal GM, Stueve T, Adamson JW (1988) Analysis of murine megakaryocyte colony size and ploidy: effects of interleukin-3. J Cell Physiol 137(3):537–544. 10.1002/jcp.1041370320 3263973 10.1002/jcp.1041370320

[34] Kaser A, Brandacher G, Steurer W et al (2001) Interleukin-6 stimulates thrombopoiesis through thrombopoietin: role in inflammatory thrombocytosis. Blood 98(9):2720–2725. 10.1182/blood.v98.9.2720 11675343 10.1182/blood.v98.9.2720

[35] Nishimura S, Nagasaki M, Kunishima S et al (2015) IL-1alpha induces thrombopoiesis through megakaryocyte rupture in response to acute platelet needs. J Cell Biol 209(3):453–466. 10.1083/jcb.201410052 25963822 10.1083/jcb.201410052 PMC4427781

[36] Machlus KR, Johnson KE, Kulenthirarajan R et al (2016) CCL5 derived from platelets increases megakaryocyte proplatelet formation. Blood 127(7):921–926. 10.1182/blood-2015-05-644583 26647394 10.1182/blood-2015-05-644583 PMC4760093

[37] Poirault-Chassac S, Six E, Catelain C et al (2010) Notch/Delta4 signaling inhibits human megakaryocytic terminal differentiation. Blood 116(25):5670–5678. 10.1182/blood-2010-05-285957 20829371 10.1182/blood-2010-05-285957

[38] Weiss-Gayet M, Starck J, Chaabouni A, Chazaud B, Morle F (2016) Notch stimulates both Self-Renewal and lineage plasticity in a subset of murine CD9High committed megakaryocytic progenitors. PLoS ONE 11(4):e0153860. 10.1371/journal.pone.0153860 27089435 10.1371/journal.pone.0153860 PMC4835090

[39] Sperling AS, Gibson CJ, Ebert BL (2017) The genetics of myelodysplastic syndrome: from clonal haematopoiesis to secondary leukaemia. Nat Rev Cancer 17(1):5–19. 10.1038/nrc.2016.112 27834397 10.1038/nrc.2016.112 PMC5470392

[40] Visconte V, Tiu RV, Rogers HJ (2014) Pathogenesis of myelodysplastic syndromes: an overview of molecular and non-molecular aspects of the disease. Blood Res 49(4):216–227. 10.5045/br.2014.49.4.216 25548754 10.5045/br.2014.49.4.216 PMC4278002

[41] Bejar R, Stevenson K, Abdel-Wahab O et al (2011) Clinical effect of point mutations in myelodysplastic syndromes. N Engl J Med 364(26):2496–2506. 10.1056/NEJMoa1013343 21714648 10.1056/NEJMoa1013343 PMC3159042

[42] Topka S, Vijai J, Walsh MF et al (2015) Germline ETV6 mutations confer susceptibility to acute lymphoblastic leukemia and thrombocytopenia. PLoS Genet 11(6):e1005262. 10.1371/journal.pgen.1005262 26102509 10.1371/journal.pgen.1005262 PMC4477877

[43] Zhang MY, Churpek JE, Keel SB et al (2015) Germline ETV6 mutations in Familial thrombocytopenia and hematologic malignancy. Nat Genet 47(2):180–185. 10.1038/ng.3177 25581430 10.1038/ng.3177 PMC4540357

[44] Noris P, Perrotta S, Seri M et al (2011) Mutations in ANKRD26 are responsible for a frequent form of inherited thrombocytopenia: analysis of 78 patients from 21 families. Blood 117(24):6673–6680. 10.1182/blood-2011-02-336537 21467542 10.1182/blood-2011-02-336537

[45] Arber DA, Orazi A, Hasserjian R et al (2016) The 2016 revision to the world health organization classification of myeloid neoplasms and acute leukemia. Blood 127(20):2391–2405. 10.1182/blood-2016-03-643544 27069254 10.1182/blood-2016-03-643544

[46] Homan CC, Scott HS, Brown AL (2023) Hereditary platelet disorders associated with germ line variants in RUNX1, ETV6, and ANKRD26. Blood 141(13):1533–1543. 10.1182/blood.2022017735 36626254 10.1182/blood.2022017735 PMC10651873

[47] Rio-Machin A, Vulliamy T, Hug N et al (2020) The complex genetic landscape of Familial MDS and AML reveals pathogenic germline variants. Nat Commun 11(1):1044. 10.1038/s41467-020-14829-5 32098966 10.1038/s41467-020-14829-5 PMC7042299

[48] Kuvardina ON, Herglotz J, Kolodziej S et al (2015) RUNX1 represses the erythroid gene expression program during megakaryocytic differentiation. Blood 125(23):3570–3579. 10.1182/blood-2014-11-610519 25911237 10.1182/blood-2014-11-610519 PMC4463808

[49] Lordier L, Bluteau D, Jalil A et al (2012) RUNX1-induced Silencing of non-muscle myosin heavy chain IIB contributes to megakaryocyte polyploidization. Nat Commun 3:717. 10.1038/ncomms1704 22395608 10.1038/ncomms1704

[50] Hock H, Meade E, Medeiros S et al (2004) Tel/Etv6 is an essential and selective regulator of adult hematopoietic stem cell survival. Genes Dev 18(19):2336–2341. 10.1101/gad.1239604 15371326 10.1101/gad.1239604 PMC522982

[51] Bluteau D, Balduini A, Balayn N et al (2014) Thrombocytopenia-associated mutations in the ANKRD26 regulatory region induce MAPK hyperactivation. J Clin Invest 124(2):580–591. 10.1172/JCI71861 24430186 10.1172/JCI71861 PMC3904625

[52] Beutler E, Yeh M, Fairbanks VF (1962) The normal human female as a mosaic of X-chromosome activity: studies using the gene for C-6-PD-deficiency as a marker. Proc Natl Acad Sci U S A 48(1):9–16. 10.1073/pnas.48.1.9 13868717 10.1073/pnas.48.1.9 PMC285481

[53] Heuser M, Thol F, Ganser A (2016) Clonal hematopoiesis of indeterminate potential. Dtsch Arztebl Int 113(18):317–322. 10.3238/arztebl.2016.0317 27215596 10.3238/arztebl.2016.0317 PMC4961884

[54] Bolton KL, Ptashkin RN, Gao T et al (2020) Cancer therapy shapes the fitness landscape of clonal hematopoiesis. Nat Genet 52(11):1219–1226. 10.1038/s41588-020-00710-0 33106634 10.1038/s41588-020-00710-0 PMC7891089

[55] Xie Z, Komrokji R, Al Ali N et al (2024) Risk prediction for clonal cytopenia: multicenter real-world evidence. Blood 144(19):2033–2044. 10.1182/blood.2024024756 38996210 10.1182/blood.2024024756 PMC11561536

[56] Fabre MA, de Almeida JG, Fiorillo E et al (2022) The longitudinal dynamics and natural history of clonal haematopoiesis. Nature 606(7913):335–342. 10.1038/s41586-022-04785-z 35650444 10.1038/s41586-022-04785-z PMC9177423

[57] Morrison SJ, Scadden DT (2014) The bone marrow niche for Haematopoietic stem cells. Nature 505(7483):327–334. 10.1038/nature12984 24429631 10.1038/nature12984 PMC4514480

[58] Raaijmakers MH, Mukherjee S, Guo S et al (2010) Bone progenitor dysfunction induces myelodysplasia and secondary leukaemia. Nature 464(7290):852–857. 10.1038/nature08851 20305640 10.1038/nature08851 PMC3422863

[59] Wang JY, Ye S, Zhong H (2017) The role of bone marrow microenvironment in platelet production and their implications for the treatment of thrombocytopenic diseases. Hematology 22(10):630–639. 10.1080/10245332.2017.1333274 28569613 10.1080/10245332.2017.1333274

[60] Ghobrial IM, Detappe A, Anderson KC, Steensma DP (2018) The bone-marrow niche in MDS and MGUS: implications for AML and MM. Nat Rev Clin Oncol 15(4):219–233. 10.1038/nrclinonc.2017.197 29311715 10.1038/nrclinonc.2017.197

[61] Tikhonova AN, Dolgalev I, Hu H et al (2019) The bone marrow microenvironment at single-cell resolution. Nature 569(7755):222–228. 10.1038/s41586-019-1104-8 30971824 10.1038/s41586-019-1104-8 PMC6607432

[62] Baccin C, Al-Sabah J, Velten L et al (2020) Combined single-cell and Spatial transcriptomics reveal the molecular, cellular and Spatial bone marrow niche organization. Nat Cell Biol 22(1):38–48. 10.1038/s41556-019-0439-6 31871321 10.1038/s41556-019-0439-6 PMC7610809

[63] Bennett JM, Catovsky D, Daniel MT et al (1982) Proposals for the classification of the myelodysplastic syndromes. Br J Haematol 51(2):189–199. https://www.ncbi.nlm.nih.gov/pubmed/6952920 6952920

[64] Harris NL, Jaffe ES, Diebold J et al (1999) The world health organization classification of neoplastic diseases of the hematopoietic and lymphoid tissues. Report of the clinical advisory committee meeting, airlie house, Virginia, November, 1997. Ann Oncol 10(12):1419–1432. 10.1023/a:1008375931236 10643532 10.1023/a:1008375931236

[65] Falini B, Martelli MP (2023) Comparison of the international consensus and 5th WHO edition classifications of adult myelodysplastic syndromes and acute myeloid leukemia. Am J Hematol 98(3):481–492. 10.1002/ajh.26812 36606297 10.1002/ajh.26812

[66] Aster JC (2023) What is in a name?? Consequences of the classification schism in hematopathology. J Clin Oncol 41(8):1523–1526. 10.1200/JCO.22.02680 36480786 10.1200/JCO.22.02680

[67] Strapatsas J, Barbulescu EC, Lauseker M et al (2021) Influence of platelet count at diagnosis and during the course of disease on prognosis in MDS patients. Ann Hematol 100(10):2575–2584. 10.1007/s00277-021-04608-7 34324021 10.1007/s00277-021-04608-7 PMC8440262

[68] Liapis K, Papadopoulos V, Pontikoglou C et al (2023) Myelodysplastic neoplasm with isolated thrombocytopenia and immune thrombocytopenic purpura in adults: insights from a comparison of two National registries. Leukemia 37(3):708–711. 10.1038/s41375-023-01819-x 36646886 10.1038/s41375-023-01819-x

[69] Al Ameri A, Jabbour E, Garcia-Manero G et al (2011) Significance of thrombocytopenia in myelodysplastic syndromes: associations and prognostic implications. Clin Lymphoma Myeloma Leuk 11(2):237–241. 10.1016/j.clml.2011.03.005 21575929 10.1016/j.clml.2011.03.005 PMC4120264

[70] IPSS-M Risk (2024)

[71] Malcovati L, Germing U, Kuendgen A et al (2007) Time-dependent prognostic scoring system for predicting survival and leukemic evolution in myelodysplastic syndromes. J Clin Oncol 25(23):3503–3510. 10.1200/JCO.2006.08.5696 17687155 10.1200/JCO.2006.08.5696

[72] Haferlach T, Nagata Y, Grossmann V et al (2014) Landscape of genetic lesions in 944 patients with myelodysplastic syndromes. Leukemia 28(2):241–247. 10.1038/leu.2013.336 24220272 10.1038/leu.2013.336 PMC3918868

[73] Nazha A, Komrokji R, Meggendorfer M et al (2021) Personalized prediction model to risk stratify patients with myelodysplastic syndromes. J Clin Oncol 39(33):3737–3746. 10.1200/JCO.20.02810 34406850 10.1200/JCO.20.02810 PMC8601291

[74] Nachtkamp K, Stark R, Strupp C et al (2016) Causes of death in 2877 patients with myelodysplastic syndromes. Ann Hematol 95:937–944 27025507 10.1007/s00277-016-2649-3

[75] Girtovitis FI, Ntaios G, Papadopoulos A, Ioannidis G, Makris PE (2007) Defective platelet aggregation in myelodysplastic syndromes. Acta Haematol 118(2):117–122 17726306 10.1159/000107653

[76] Fröbel J, Cadeddu R-P, Hartwig S et al (2013) Platelet proteome analysis reveals integrin-dependent aggregation defects in patients with myelodysplastic syndromes. Mol Cell Proteom 12(5):1272–1280 10.1074/mcp.M112.023168 PMC3650338 23382103

[77] Levy JH, Neal MD, Herman JH (2018) Bacterial contamination of platelets for transfusion: strategies for prevention. Crit Care 22(1):271. 10.1186/s13054-018-2212-9 30367640 10.1186/s13054-018-2212-9 PMC6204059

[78] Bryan J, Jabbour E, Prescott H, Kantarjian H (2010) Thrombocytopenia in patients with myelodysplastic syndromes. Seminars in hematology: Elsevier;:274–280 10.1053/j.seminhematol.2010.02.006 PMC4428284 20620439

[79] Panch SR, Guo L, Vassallo R (2023) Platelet transfusion refractoriness due to HLA alloimmunization: evolving paradigms in mechanisms and management. Blood Rev 62:101135. 10.1016/j.blre.2023.101135 37805287 10.1016/j.blre.2023.101135

[80] Mo A, Wood E, Shortt J, Hu E, McQuilten Z (2023) Platelet transfusions and predictors of bleeding in patients with myelodysplastic syndromes. Eur J Haematol 111(4):592–600. 10.1111/ejh.14049 37452616 10.1111/ejh.14049 PMC10952506

[81] https://www.redcross.org/about-us/news-and-events/press-release/2024/red-cross-declares-emergency-blood-shortage-calls-for-donations-during-national-blood-donor-month.html. American Red Cross, Accessed in Feburary, (2024)

[82] Okamoto S, Hijikata-Okunomiya A, Wanaka K, Okada Y, Okamoto U (1997) Enzyme-controlling medicines: introduction. Semin Thromb Hemost 23(6):493–501. 10.1055/s-2007-996127 9469621 10.1055/s-2007-996127

[83] Vijenthira A, Premkumar D, Callum J et al (2019) The management and outcomes of patients with myelodysplastic syndrome with persistent severe thrombocytopenia: an observational single centre registry study. Leuk Res 76:76–81. 10.1016/j.leukres.2018.12.002 30580105 10.1016/j.leukres.2018.12.002

[84] Mo A, Weinkove R, Wood EM et al (2024) Use of platelet transfusions and Tranexamic acid in patients with myelodysplastic syndromes: A clinical practice survey. Eur J Haematol 112(4):621–626. 10.1111/ejh.14156 38123137 10.1111/ejh.14156

[85] Li J, Yang C, Xia Y et al (2001) Thrombocytopenia caused by the development of antibodies to thrombopoietin. Blood 98(12):3241–3248. 10.1182/blood.v98.12.3241 11719360 10.1182/blood.v98.12.3241

[86] Clemons Bankston P, Al-Horani RA (2019) New small molecule drugs for thrombocytopenia: chemical, Pharmacological, and therapeutic use considerations. Int J Mol Sci 20(12). 10.3390/ijms20123013 10.3390/ijms20123013 PMC6628068 31226783

[87] Kuter DJ, Bussel JB, Lyons RM et al (2008) Efficacy of Romiplostim in patients with chronic immune thrombocytopenic purpura: a double-blind randomised controlled trial. Lancet 371(9610):395–403. 10.1016/S0140-6736(08)60203-2 18242413 10.1016/S0140-6736(08)60203-2

[88] Bussel JB, Cheng G, Saleh MN et al (2007) Eltrombopag for the treatment of chronic idiopathic thrombocytopenic purpura. N Engl J Med 357(22):2237–2247. 10.1056/NEJMoa073275 18046028 10.1056/NEJMoa073275

[89] Jurczak W, Chojnowski K, Mayer J et al (2018) Phase 3 randomised study of Avatrombopag, a novel thrombopoietin receptor agonist for the treatment of chronic immune thrombocytopenia. Br J Haematol 183(3):479–490. 10.1111/bjh.15573 30191972 10.1111/bjh.15573 PMC6282556

[90] Peck-Radosavljevic M, Simon K, Iacobellis A et al (2019) Lusutrombopag for the treatment of thrombocytopenia in patients with chronic liver disease undergoing invasive procedures (L-PLUS 2). Hepatology 70(4):1336–1348. 10.1002/hep.30561 30762895 10.1002/hep.30561 PMC6849531

[91] Shin J, Kim MJ, Quan X et al (2023) Thrombopoietin receptor agonist antibody for treating chemotherapy-induced thrombocytopenia. BMC Cancer 23(1):490. 10.1186/s12885-023-10975-3 37259024 10.1186/s12885-023-10975-3 PMC10230746

[92] Kantarjian H, Fenaux P, Sekeres MA et al (2010) Safety and efficacy of Romiplostim in patients with lower-risk myelodysplastic syndrome and thrombocytopenia. J Clin Oncol 28(3):437–444. 10.1200/JCO.2009.24.7999 20008626 10.1200/JCO.2009.24.7999

[93] Kubasch AS, Giagounidis A, Metzgeroth G et al (2022) Prospective validation of a biomarker-driven response prediction model to Romiplostim in lower-risk myelodysplastic neoplasms - results of the EUROPE trial by EMSCO. Leukemia 36(10):2519–2527. 10.1038/s41375-022-01669-z 36071100 10.1038/s41375-022-01669-z PMC9522582

[94] Wang ES, Lyons RM, Larson RA et al (2012) A randomized, double-blind, placebo-controlled phase 2 study evaluating the efficacy and safety of Romiplostim treatment of patients with low or intermediate-1 risk myelodysplastic syndrome receiving Lenalidomide. J Hematol Oncol 5:71. 10.1186/1756-8722-5-71 23190430 10.1186/1756-8722-5-71 PMC3520696

[95] Greenberg PL, Garcia-Manero G, Moore M et al (2013) A randomized controlled trial of Romiplostim in patients with low- or intermediate-risk myelodysplastic syndrome receiving decitabine. Leuk Lymphoma 54(2):321–328. 10.3109/10428194.2012.713477 22906162 10.3109/10428194.2012.713477

[96] Kantarjian HM, Giles FJ, Greenberg PL et al (2010) Phase 2 study of Romiplostim in patients with low- or intermediate-risk myelodysplastic syndrome receiving Azacitidine therapy. Blood 116(17):3163–3170. 10.1182/blood-2010-03-274753 20631375 10.1182/blood-2010-03-274753 PMC3324162

[97] Vicente A, Patel BA, Gutierrez-Rodrigues F et al (2020) Eltrombopag monotherapy can improve hematopoiesis in patients with low to intermediate risk-1 myelodysplastic syndrome. Haematologica 105(12):2785–2794. 10.3324/haematol.2020.249995 33256377 10.3324/haematol.2020.249995 PMC7716353

[98] Comont T, Meunier M, Cherait A et al (2021) Eltrombopag for myelodysplastic syndromes or chronic myelomonocytic leukaemia with no excess blasts and thrombocytopenia: a French multicentre retrospective real-life study. Br J Haematol 194(2):336–343. 10.1111/bjh.17539 34151423 10.1111/bjh.17539

[99] Roth M, Will B, Simkin G et al (2012) Eltrombopag inhibits the proliferation of leukemia cells via reduction of intracellular iron and induction of differentiation. Blood 120(2):386–394. 10.1182/blood-2011-12-399667 22627766 10.1182/blood-2011-12-399667 PMC3398759

[100] Kalota A, Selak MA, Garcia-Cid LA, Carroll M (2015) Eltrombopag modulates reactive oxygen species and decreases acute myeloid leukemia cell survival. PLoS ONE 10(4):e0126691. 10.1371/journal.pone.0126691 25915523 10.1371/journal.pone.0126691 PMC4411049

[101] Svensson T, Chowdhury O, Garelius H et al (2014) A pilot phase I dose finding safety study of the thrombopoietin-receptor agonist, Eltrombopag, in patients with myelodysplastic syndrome treated with Azacitidine. Eur J Haematol 93(5):439–445. 10.1111/ejh.12383 24853277 10.1111/ejh.12383

[102] Platzbecker U, Wong RS, Verma A et al (2015) Safety and tolerability of Eltrombopag versus placebo for treatment of thrombocytopenia in patients with advanced myelodysplastic syndromes or acute myeloid leukaemia: a multicentre, randomised, placebo-controlled, double-blind, phase 1/2 trial. Lancet Haematol 2(10):e417–e426. 10.1016/S2352-3026(15)00149-0 26686043 10.1016/S2352-3026(15)00149-0

[103] Mittelman M, Platzbecker U, Afanasyev B et al (2018) Eltrombopag for advanced myelodysplastic syndromes or acute myeloid leukaemia and severe thrombocytopenia (ASPIRE): a randomised, placebo-controlled, phase 2 trial. Lancet Haematol 5(1):e34–e43. 10.1016/S2352-3026(17)30228-4 29241762 10.1016/S2352-3026(17)30228-4

[104] Kim N, Norsworthy KJ, Subramaniam S et al (2022) FDA approval summary: decitabine and Cedazuridine tablets for myelodysplastic syndromes. Clin Cancer Res 28(16):3411–3416. 10.1158/1078-0432.CCR-21-4498 35435961 10.1158/1078-0432.CCR-21-4498 PMC9378483

[105] Diesch J, Zwick A, Garz AK, Palau A, Buschbeck M, Gotze KS (2016) A clinical-molecular update on azanucleoside-based therapy for the treatment of hematologic cancers. Clin Epigenetics 8:71. 10.1186/s13148-016-0237-y 27330573 10.1186/s13148-016-0237-y PMC4915187

[106] Fenaux P, Mufti GJ, Hellstrom-Lindberg E et al (2009) Efficacy of Azacitidine compared with that of conventional care regimens in the treatment of higher-risk myelodysplastic syndromes: a randomised, open-label, phase III study. Lancet Oncol 10(3):223–232. 10.1016/S1470-2045(09)70003-8 19230772 10.1016/S1470-2045(09)70003-8 PMC4086808

[107] Lubbert M, Suciu S, Baila L et al (2011) Low-dose decitabine versus best supportive care in elderly patients with intermediate- or high-risk myelodysplastic syndrome (MDS) ineligible for intensive chemotherapy: final results of the randomized phase III study of the European organisation for research and treatment of Cancer leukemia group and the German MDS study group. J Clin Oncol 29(15):1987–1996. 10.1200/JCO.2010.30.9245 21483003 10.1200/JCO.2010.30.9245

[108] Jung HA, Maeng CH, Kim M, Kim S, Jung CW, Jang JH (2015) Platelet response during the second cycle of decitabine treatment predicts response and survival for myelodysplastic syndrome patients. Oncotarget 6(18):16653–16662. 10.18632/oncotarget.3914 25938546 10.18632/oncotarget.3914 PMC4599296

[109] Baumann J, Spindler M, Throm Y, Lubbert M, Bender M (2022) Absence of early platelet increment in healthy mice during decitabine treatment. Sci Rep 12(1):22266. 10.1038/s41598-022-26821-8 36564544 10.1038/s41598-022-26821-8 PMC9789030

[110] Garcia-Manero G, Santini V, Almeida A et al (2021) Phase III, randomized, Placebo-Controlled trial of CC-486 (Oral Azacitidine) in patients with Lower-Risk myelodysplastic syndromes. J Clin Oncol 39(13):1426–1436. 10.1200/JCO.20.02619 33764805 10.1200/JCO.20.02619 PMC8099416

[111] Garcia JS, Platzbecker U, Odenike O et al (2024) Efficacy and safety of venetoclax plus Azacitidine for patients with Treatment-Naive High-Risk myelodysplastic syndromes. Blood. 10.1182/blood.2024025464 39652823 10.1182/blood.2024025464 PMC11923426

[112] Meng F, Chen X, Yu S et al (2020) Safety and efficacy of Eltrombopag and Romiplostim in myelodysplastic syndromes: A systematic review and Meta-Analysis. Front Oncol 10:582686. 10.3389/fonc.2020.582686 33324559 10.3389/fonc.2020.582686 PMC7727449

[113] Saito S, Ota S, Seshimo H et al (2002) Platelet transfusion refractoriness caused by a mismatch in HLA-C antigens. Transfusion 42(3):302–308. 10.1046/j.1537-2995.2002.00051.x 11961234 10.1046/j.1537-2995.2002.00051.x

